# What factors are most relevant to the assessment of work ability of employees on long-term sick leave? The physicians’ perspective

**DOI:** 10.1007/s00420-012-0783-3

**Published:** 2012-05-24

**Authors:** Patricia M. Dekkers-Sánchez, Haije Wind, Judith K. Sluiter, Monique H. W. Frings-Dresen

**Affiliations:** 1 Coronel Institute of Occupational Health, Academic Medical Center, University of Amsterdam, P.O. Box 22700, 1100 DE Amsterdam, The Netherlands; 2 Research Center for Insurance Medicine, Academic Medical Center, University of Amsterdam, Amsterdam, The Netherlands

**Keywords:** Long-term sick leave, Sick-listed employees, Work ability assessment, Physicians’ perspective, Disability assessment

## Abstract

**Purpose:**

To reach insurance physician (IPs) consensus on factors that must be taken into account in the assessment of the work ability of employees who are sick-listed for 2 years.

**Methods:**

A Delphi study using online questionnaires was conducted from October 2010 to March 2011.

**Results:**

One hundred and two insurance physicians reached a consensus on important factors for return to work (RTW) of employees on long-term sick leave; from those factors, the most relevant for the assessment of work ability was determined. From a total of 22 relevant factors considered for the return to work of long-term sick-listed employees, consensus was reached on nine relevant factors that need to be taken into account in the assessment of the work ability of employees on long-term sick leave. Relevant factors that support return to work are motivation, attitude towards RTW, assessment of cognitions and behaviour, vocational rehabilitation in an early stage and instruction for the sick-listed employee to cope with his disabilities. Relevant factors that hinder RTW are secondary gain from illness, negative perceptions of illness, inefficient coping style and incorrect advice of treating physicians regarding RTW.

**Conclusions:**

Non-medical personal and environmental factors may either hinder or promote RTW and must be considered in the assessment of the work ability of long-term sick-listed employees. Assessment of work ability should start early during the sick leave period. These factors may be used by IPs to improve the quality of the assessment of the work ability of employees on long-term sick leave.

## Introduction

Long-term sick leave is a recognised major health problem (Henderson et al. 2005), and many industrialised countries have high percentages of people who are unproductive and who claim work disability benefits for medical reasons (Black 2008; OECD 2010). Employees on sick leave need specific guidance to prevent them from being sick-listed for the long term and from requiring long-term disability benefits. The correct assessment of the sick-listed employees’ ability to work is crucial to enhance the return to work; apparently, however, physicians lack sufficient knowledge about the proper assessment of workers on sick leave and the management of their return to work (e.g. Elms et al. 2005; Pransky et al. 2002; Soklaridis et al. 2011; Wahlstrőm and Alexanderson 2004). For example, although management of work-related disability and absence due to illness is an essential part of the work of occupational health professionals, previous research has shown that assessing the disability, monitoring and advising during sickness absence are considered to be of low priority by occupational physicians (Macdonald et al. 2000). In contrast, the assessment of the ability to work was determined to be important by both employers and employees (Reetoo et al. 2005).

The category of physicians who evaluate patients’ ability to work and who assist them in returning to work varies by country. In some countries, the assessment of the functional ability to RTW of employees on sick leave is performed by general practitioners, family physicians, occupational physicians, insurance physicians, primary care practitioners, specialists or other physicians. In the Netherlands, sick-listed employees between 18 and 65 years of age who are unable to work due to medical reasons and who meet the eligibility requirements can apply for a disability pension after a period of 1.5 years of absence due to illness. After 2 years of sick leave, employees undergo an assessment to determine their work ability, which includes an assessment of their medical condition, functional limitations, working capacity and prognosis regarding impairments, limitations on activity and ability to resume work.

Insurance physicians (IPs) are responsible for the medical assessment of the work ability of employees on sick leave in the Netherlands. These medical professionals follow a 4-year in-company training before they can be officially recognised as registered (board certified) insurance physicians. To gain insight into the factors that either impede or promote the return to work of long-term sick-listed employees, we investigated the opinions of registered insurance physicians because they specialise in the assessment of the work ability of employees on long-term sick leave and may be regarded as experts in the field based on their specific expertise.

In this Delphi study, we refer to the assessment of work ability of employees on 2-years sick leave, according to the regulations of the Dutch legislation (Work and Incoming Act 2005). The Work and Incoming Act 2005 has two aims: to promote reintegration and to protect the income of workers who are work disabled due to illness. The primary aim of this legislation is to promote work resumption, increasing the reintegration of employees with health-related work restrictions (OECD 2007). Taking into account this legislation, the assessment of work disability should also be directed to RTW instead of focusing purely on the physical and/or mental capacity to perform work.

The available literature on RTW and sick leave has been focused mainly on the determinants of the return to work of employees on short-term sick leave, while largely ignoring the importance of the determinants of long-term sick leave. Literature shows that there is no international consensus about the definition of long-term sick leave and short-term sick leave. In the present study, we define long-term sick leave as sickness absence during at least 1.5 years. A systematic review showed that most studies on sick leave are based on sickness absence periods of 6 weeks or less, and there is much less literature about sick leave periods longer than 6 weeks (Dekkers-Sánchez et al. 2008).

The importance of early work resumption for employees on sick leave has been highlighted by several previous studies (e.g. Bernacki et al. 2000; Tveito et al. 2004). The literature suggests that the impact of factors related to sick leave and absence from work can vary through the different stages of illness (Krause et al. 2001; Burton et al. 2003). The initial onset of absence from work is almost always due to medical reasons. Sufficient evidence suggests that both medical and non-medical factors play a role in the maintenance of sick leave (Dekkers-Sánchez et al. 2008). This diversity of factors could explain why the resumption of work is increasingly difficult as the time absent from work increases (WHO 2003). Despite the importance of long-term sickness absence, previous research has shown that there is a lack of scientific knowledge on the factors associated with long-term sick leave (Dekkers-Sánchez et al. 2008).

Literature shows that the causes of long-term sick leave and complex may involve medical, psychosocial, financial, organisational and work-related factors (Alexanderson 2004). Therefore, a proper workability assessment should take into account all factors that seem responsible for the maintenance of the sickness absence. After 2 years of sick leave, these complex conditions require a multifactorial analysis, including the medical situation, work situation and personal situation of the claimant. This implies that the assessment of workability should include not only the medical factors, but also the non-medical factors responsible for a decreased ability to perform work. With better knowledge about the factors associated with sickness absence, IPs can make useful recommendations to achieve RTW, which is in concordance with the Dutch legislation, aiming at improving RTW outcomes.

Despite the important role of physicians in the RTW process, little is known about the views of physicians on the factors that should be addressed in the evaluation of the work ability of employees on long-term sick leave. Therefore, enhancing the knowledge of physicians regarding these relevant factors is warranted.

The aim of this study was to determine the most relevant factors that should be addressed during the assessment of the work ability of sick-listed employees.

The following specific question was addressed:

Which relevant factors, according to insurance physicians, should be taken into account during the assessment of the work ability of employees who are on sick leave for 2 years?

## Methods

We used the Delphi technique, an iterative group process of multi-round questionnaires, with the aim of gaining a consensus from a panel of experts on a particular issue (e.g. Jones and Hunter 1995; Black 2006).

### Participants

The participants were selected from the population of insurance physicians working at the Employee Benefits Insurance Authority (UWV), an organisation that employs the largest number of insurance physicians in the Netherlands. Purposive sampling was employed to recruit experienced insurance physicians from all different geographical regions within the Netherlands. The potential participants were contacted through their work email addresses. Information about the study was sent by email to all IPs working at the organisation with experience in the assessment of the work ability of employees on long-term sick leave. Subjects who were eligible for this study included registered insurance physicians with experience in the medical assessment of employees on sick leave for more than 1.5 years. The other eligibility criteria were that physicians were willing to take part in four Delphi rounds and were interested in sharing their views. All potential participants who met the study criteria were invited to enrol themselves by sending an email to the researchers. Our selection criteria aimed to ensure an adequate breadth of expertise and a variety of perspectives on factors related to long-term sick leave and to ensure the availability of the selected people within the time frame of the study. Eligible subjects received written information concerning the aims and procedures of the study.

### Procedure

The electronic Delphi method was used to reach an agreement on factors that should be addressed during the assessment of the work ability of employees on long-term sick leave. Before starting the study, a pilot study was performed on a small group of IPs not involved in the Delphi process (*n* = 5) to ensure that there was common understanding of the questions. The panellists did not know who else was participating in the Delphi study or the answers that the other panellists gave. The study comprised two preliminary rounds and two main rounds.

### Preliminary rounds

The aim of the two preliminary rounds of this study was to collect the input for the main rounds. The panellists achieved consensus on important factors that either hinder or promote RTW by employees on long-term sick leave. These factors were then presented to the panellists during the main rounds.

A preliminary questionnaire was developed and administered to the participants via a link to the questionnaire with corresponding instructions contained in an email. We used structured questions with the “relevant/not relevant” answer format. Additionally, we asked the panellists some background questions such as gender, age and years of experience as an IP. In every round, the panellists had 2 weeks to respond, and reminders were sent out 7 days before the deadline. Data were analysed after each round to generate a list of factors for subsequent rounds. Factors that were identified by over 80 % of study participants in the preliminary rounds were resubmitted in the following rounds. This procedure allowed us to reduce the original list of factors to those that were most relevant.

### First preliminary round

We developed a structured questionnaire based on previous study results for the first preliminary round. The factors included in the preliminary rounds were compiled from three sources: (1) a systematic review of factors commonly associated with long-term sick leave (Dekkers-Sánchez et al. 2008); (2) a focus group study on the patients’ perspectives on factors related to long-term sick leave (Dekkers-Sánchez et al. 2010); and (3) a qualitative study on the views of vocational rehabilitation professionals on factors that contribute to successful RTW (Dekkers-Sánchez et al. 2011). The panellists were also encouraged to add additional factors based on their clinical experience.

Appendix 1 contains the preliminary list that includes 23 factors that hinder and 28 factors that promote RTW, which was incorporated into the first preliminary round.

### Second preliminary round

The second preliminary questionnaire comprised additional “new factors” (*n* = 35) included by the panellists and that were identified in the first preliminary round. The panellists were asked the question: *Which of the following new factors mentioned by your colleagues are, according to your experience, important for RTW of long*-*term sick listed employees?* The respondents were asked to score each individual factor as either important or not important. As in the first preliminary round, factors selected by at least 80 % of the panellists were included in the questionnaire in the first main round.

### Main rounds

The aim of the main rounds was to identify the factors that should be included in the assessment of the work ability of employees on long-term sick leave according to the panellists.

### First main round

In this round, the panellists were asked to judge whether each of the factors included on the questionnaire were either relevant or irrelevant to the assessment of work ability according to their experience. We asked the IPs: *Which of the following factors are, in your opinion, relevant to the assessment of the workability of long*-*term sick listed employees?* The input for the first main round comprised a list of 51 factors that resulted from the preliminary round questionnaires. The answer format was relevant/not relevant. Only the factors mentioned by at least 80 % of the respondents and additional new factors included by individual panellists during the preliminary rounds were used to populate this questionnaire.

### Second main round

The aim of the last round was to identify the most relevant factors for the assessment of the work ability of employees on long-term sick leave. The factors mentioned by at least 80 % of the panellists in the previous round were included in the last questionnaire. We presented the final list of twenty-two relevant factors to the panellists and asked them to select ten factors that, in their opinion, must be taken into account during the assessment of the work ability of employees who are sick-listed for 2 years. The format for this round of questions was a checkbox list. We asked the IPs: *Please select from* *the following relevant factors ten factors that in your opinion, definitely need to be included in the assessment of the work ability of long*-*term sick-listed employees.*

### Data analysis

#### Preliminary rounds

After the first preliminary round, a content analysis of the newly added factors was performed. Only new factors were included in the subsequent round.

A quantitative analysis of the responses was performed after the preliminary rounds. Data from the questionnaires were stored in SPSS 18. Incomplete questionnaires were not used. Consensus was defined as a “general agreement of a substantial majority”. The following a priori criterion was used to determine the level of consensus: consensus was defined as having been achieved if 80 % or more of the panel members rated that factor as “important”. Socio-demographic data were compiled after each round and analysed using descriptive statistics (e.g. frequencies, mean/median and standard-deviation).

### Main rounds

A quantitative analysis of the responses was performed after the main rounds. In the first main round, consensus was defined as having been achieved if 80 % or more of the panel members rated that factor as “relevant”. In the second main round, the factors selected by at least 55 % of the panellists were included in the final list of factors. These factors comprised the final list of relevant factors for the assessment of the work ability of employees on long-term sick leave.

## Results

The studies were performed during a 4-month period, from November 2010 until March 2011.

### Participants

A total of 194 insurance physicians were initially contacted to be part of the expert panel. A total of 108 (55 %) of these IPs agreed to participate and were included in the mailing list. Eighty-six IPs did not respond to the invitation to take part of the study, giving no reason for non-participation. Only registered IPs with experience in the assessment of employees on sick leave for 2 years were included in the sample. Of those 108 willing respondents, 107 completed the first round (99 %), 105 (97 %) completed the second round, 103 (95 %) completed the third round and 102 (94 %) completed the final round. The final round sample (*n* = 102) included 50 women and 52 men, and their ages varied from 32 to 64 years. All included participants were registered IPs working within the Netherlands. The experience of the study participants as insurance physicians varied between 7 and 33 years.

### Results of the preliminary rounds

From a total of 56 factors, 32 factors were agreed upon by at least 80 % of the participants. The qualitative analysis of the new factors included by the participants generated 35 additional factors. In the second preliminary round, the 35 new factors were returned to the participants who were then asked to choose those factors that are important for RTW. More than 80 % of the panellists found 22 of the new factors important. The result of the two preliminary rounds was a list of 54 factors.

### Results of the main rounds

First main round: From among 54 factors, 22 relevant factors for RTW for the assessment of work ability were mentioned by at least 80 % of the participants. See Appendix 2 and 3 for factors that either hinder or promote RTW of long-term sick-listed employees.

Second main round: More than 55 % of the participants determined that nine of the 22 relevant factors should be a part of the work ability assessment of employees on sick leave. See Table 1 for the 9 relevant factors determined to be important for the assessment of work ability.

**Table 1:** Factors that should be included in the assessment of the work ability of employees on long-term sick leave according insurance physicians

Factors that promote RTW	(%)	Factors that hinder RTW	(%)
Motivation of sick-listed employee to RTW	79	Secondary gain from illness	76
Positive attitude of employee towards resuming work	75	Inefficient coping style	70
Providing RTW vocational rehabilitation as soon as possible	70	Incorrect advice of treating physicians regarding RTW	69
Assessment of cognitions and behaviour	64	Negative illness perceptions	57
Teaching the sick-listed employee to cope with his/her disabilities	60		

## Discussion

### Summary of main findings

Insurance physicians reached a consensus on nine relevant factors for RTW that must be taken into account in the assessment of the work ability of employees on long-term sick leave: work motivation, attitude towards RTW, changing inadequate cognitions and behaviour, early vocational rehabilitation, learning how to cope with disabilities, secondary gain from illness, negative illness perceptions, inefficient coping style and incorrect advice of treating physicians regarding RTW.

Our findings point to the importance of obtaining a complete picture of the situation of employees on long-term sick leave during the period of work ability assessment. This result implies that, in addition to an understanding of the medical condition, information about non-medical factors is necessary for a proper assessment of the work ability of employees on long-term sick leave. To the best of our knowledge, this is the first study that focuses on relevant factors independent of the primary diagnosis to be used in the assessment of the work ability of chronic work-disabled employees. The results of the present study may be particularly useful for physicians involved in RTW cases, and it may serve as another tool to be used in the assessment of the work ability of employees suffering from chronic conditions. The results allow us to recommend a quality improvement approach for the assessment of the work ability of employees on long-term sick leave. The identified factors could be the basis for a tool to guide physicians in the assessment of work ability of employees on long-term sick leave.

The assessment of work ability by IP’s is primarily focused on the actual workability of the employee in terms of physical and/or mental capacity to perform work. The identification of the factors that maintain disability and the factors that promote work resumption contributes to make a complete investigation of the actual situation of a claimant and his ability to perform work. We believe that increasing the awareness of IP’s about the relevance of these factors in their context could improve the quality of the assessment of workability of employees on long-term sick leave. The identification of factors that hinder or promote work resumption during the assessment of workability could enhance the quality of the assessment of workability. In order to facilitate insight of the IPs into the complex factors related to work disability, we used the model perpetuating factors for long-term sick leave and promoting factors for return to work to classify the factors in the Delphi study (Dekkers-Sánchez et al. 2010).

In the second preliminary round, the participants were asked to mention which factors they considered important for RTW. The IPs mentioned 22 important factors for RTW. In the first main round, IPs were asked to choose the most relevant factors for the assessment of workability from these 22 important factors for RTW. Nine important factors for RTW were mentioned as the most relevant factors for the assessment of workability.

The aim of the present study was to obtain consensus about relevant factors that should be taken into account during the assessment of workability of employees on long-term sick leave. In the last rounds of the Delphi study, the important factors for RTW mentioned by the participants were linked to the assessment of workability. Attention for factors related to RTW is consistent with the aim of the Dutch legislation, Work and Incoming Act 2005, aiming at enhancing work participation of employees on long-term sick leave (OECD 2007). Sufficient evidence shows that both medical and non-medical factors contribute to a decreased ability to perform work. Dutch IPs found that nine relevant factors should be included in the assessment of employees on long-term sick leave. With better knowledge about the factors associated with sickness absence, IPs can make a completer assessment and make useful recommendations to achieve RTW, which is in concordance with the Dutch legislation, aiming at improving RTW outcomes.

In the last main round of questionnaires, the majority of the panellists (>55 %) mentioned that factors related to cognition and behaviour (motivation to RTW, secondary gain from illness, positive attitude towards RTW, inefficient coping style and negative illness perceptions) must be considered in the assessment of the work ability of employees on long-term sick leave. This result is consistent with previous studies on factors associated with long-term sick leave. An early study of employees on sick leave for 2 years also showed that both negative perceptions of illness and inefficient coping style hindered RTW (Dekkers-Sánchez et al. 2010). Another study on the views of vocational rehabilitation professionals found that positive cognition, work motivation and positive attitude of the sick-listed employee regarding RTW promoted work resumption of employees on long-term sick leave (Dekkers-Sánchez et al. 2011). An important finding is that the results of these previous studies show that sick-listed employees, vocational rehabilitation professionals and insurance physicians agree that motivation, inefficient coping style, negative illness perceptions and positive attitude towards work resumption are relevant factors that either promote or hinder RTW. Interestingly, three of the nine relevant factors for the assessment of work ability (secondary gain from illness, instruction for the sick-listed employee to cope with his disabilities and incorrect advice from treating physicians concerning RTW) were mentioned by insurance physicians but were not mentioned by the sick-listed employees of the vocational rehabilitation professionals as being relevant factors for RTW.

Obstacles for RTW may consist of a combined interaction between medical, psychosocial and environmental factors (Dekkers-Sánchez et al. 2010). Negative beliefs about work during a period of absence due to illness may decrease the work rehabilitation efforts and the motivation to RTW of the sick-listed employee. Negative beliefs can also elicit avoiding behaviour, such as staying sick longer than necessary, as a way of dealing with physical or psychological complaints or other psychosocial problems. Negative thoughts and associated behaviours may thus hinder recovery and promote further sick leave. According to the findings of the present study, we can conclude that factors related to thoughts, behaviours and environmental factors seem to play a crucial role in the development of chronic work disability and should therefore be considered during the assessment of the work ability of employees on long-term sick leave. One remarkable finding was that functional limitations and handicaps due to disease were not mentioned by the majority of our panellists as factors that hinder RTW of employees on long-term sick leave. This result is consistent with the assumption that factors related to RTW may change over time (Krause et al. 2001) and that the development of chronicity and incapacity is often more dependent on psychosocial than on medical factors (e.g. Stephens et al. 2002). This fact could explain why health status is no longer the primary factor in sick leave after 2 years, which is consistent with the observations of the current study as well.

Literature shows that some of the factors mentioned by the experts in the present study have also been mentioned in quantitative studies on factors related to sickness absence spells shorter than 1.5 years. It must be noted that most quantitative studies on these relevant factors are not focused on absence spells of 1.5 years of more. This is concordance with the findings in a systematic review on factors associated with long-term sick leave in sick-listed employees (Dekkers-Sánchez et al. 2008). Quantitative studies on the relevant factors associated with sick leave longer than 1.5 years are needed to confirm our findings.

### Methodological considerations

The electronic Delphi technique we used proved to be a feasible, time- and cost-efficient method. A strength of this study is that we elicited the views of a wide range of experts that covered a broad representation of views.

Although the Delphi method has been widely used in health research, studies using the Delphi technique have some variability in their methodology (Sinha et al. 2011). In the present study, consensus was defined as an agreement of at least 80 % (Piram et al. 2011). In the last round, we decided that factors selected by a majority of panellists would be included in the final list, and 55 % can thus be accepted as a majority (Slebus et al. 2008). Some authors have suggested that the use of a structured questionnaire in the first round, instead of an open-ended questionnaire, may restrict the ability of the experts to respond to the original question (Thompson 2009). In the first questionnaire, we used a preliminary list of factors generated in previous studies, but we also encouraged participants to add new factors to the preliminary list. This method ensured that we did not overlook any important factors, and it allowed us to elicit 35 new factors that were incorporated in the subsequent questionnaire. Other studies have also used this pragmatic approach successfully (e.g. Payne et al. 2007; Dionne et al. 2008).

This study makes a unique contribution in several ways. First, the study increased our understanding of important factors that should be considered in the assessment of the work ability of employees on long-term sick leave and that are independent of the diagnosis. Second, it covers, from the physicians’ perspective, a breadth of factors associated with RTW of employees on long-term sick leave. Third, it is based on a large and heterogeneous sample of experts from all geographical regions in the country, with different demographics and varying experience with employees suffering from all types of medical complaints. Fourth, the sample reflects the characteristics of the population of IPs in the Netherlands because it was drawn from an employees’ compensation organisation that covers 95 % of the working population of insurance physicians in the country. Fifth, our panellists can be regarded as experts in the field of assessment of the work ability of employees on long-term sick leave due to their specific and extensive expertise on this topic.

### Implications for clinical practice and future research

The results of this study suggest that after 2 years of sick leave, the focus of physicians should shift from a strictly disease-oriented approach to an individual and context-oriented approach to identify the factors that hinder recovery and encourage work resumption. Extending their focus to non-medical factors could enable physicians to target specific obstacles to work resumption and to adapt their advice to help sick workers to remain at work or to get back to work more quickly after a period of illness. The identification by health professionals of factors that hinder or promote RTW at an earlier stage of sick leave, preferably not later than the first 3 months of sick leave, and the implementation of strategies and interventions targeting these factors could help decrease the chance of developing chronic work disability.

Although we gained valuable insight into factors that are relevant for RTW that should be addressed by the assessment of work ability of long-term sick-listed employees, future studies should determine whether these factors occur frequently and whether they affect RTW outcomes. The results represent the consensus of experts in this field and will be used to design a tool to support the medical assessment of the work ability of employees on long-term sick leave.

We expect that the results of the present study will improve the overall quality of the assessment of the work ability and subsequent guidance of sick-listed employees by emphasising the importance of taking into account non-medical factors.

The relation between thoughts and RTW is an important finding, as some factors related to thoughts and beliefs are potentially amenable to change, which offers possibilities for the improvement of work participation of employees on long-term sick leave. These findings suggest that the employees’ thoughts and behaviour regarding RTW may be at least as important as the medical condition of the sick-listed employee, especially in chronic conditions. Acknowledging and addressing factors such as lack of motivation, negative attitude towards RTW, negative illness perceptions and secondary gain issues is required to assess work ability accurately. Early RTW interventions targeting thoughts and behaviour at earlier stages of sick leave, preferably not later than after 3 months of sick leave, could also be beneficial for employees on long-term sick leave due to other types of complaints.

Specific skills training for physicians to learn to recognise these obstacles and motivators for RTW could improve the quality of guidance for employees on sick leave, for example, by providing tailor-made advice or by referring sick-listed employees to specific behavioural or mental health practitioners as needed. Promoting factors such as beginning RTW rehabilitation early, influencing thoughts/behaviour/motivation and teaching the employee to cope with his disabilities can provide excellent ways to accomplish successful vocational rehabilitation. It is interesting to note that in previous research, both patients on long-term sick leave (Dekkers-Sánchez et al. 2010) and vocational rehabilitation, professionals [Dekkers-Sánchez et al. 2011) mentioned that an early start to work rehabilitation, motivation and attitude of the sick-listed employee and instruction on how to cope with disabilities were important promoting factors for RTW.

The assessment of non-medical factors could be used to select sick-listed employees who may potentially benefit from early RTW interventions and may help reduce chronic work disability. Future research on early RTW-focused interventions, preferably starting not later than the first 3 months of the sick leave period and that target specific factors that hinder or promote RTW, may offer promising ways to achieve early work resumption of employees on long-term sick leave.

According to the panellists, factors related to the individual such as motivation, positive attitude towards RTW, assessment of cognitions and behaviour, an early start to vocational rehabilitation in an early stage and instruction for the sick-listed employee to cope with his disability promote RTW and should be considered in the evaluation of work ability. Barriers for RTW that also should be addressed in the assessment of work ability are inefficient coping strategies, secondary gain from illness, negative illness perceptions and inadequate advice from treating physicians. Experienced IPs agreed that non-medical barriers and factors that promote RTW should be taken into account in the assessment of the work ability of employees on long-term sick leave.

## Appendix 1

See Table 2.

**Table 2:** Preliminary list of 51 factors that either hinder or promote RTW based on previous study results and that were included in the first Delphi questionnaire

Factors that promote RTW	Factors that hinder RTW
Motivation of sick-listed employee to RTW	Presence of disease
Financial consequences of sick leave	Activity limitations
Positive self-efficacy expectations	Participation restrictions
Degree of control over working situation	Negative environmental factors
Positive attitude of employee towards work resumption	Older age
Effective communication with employee	Low educational level
Increasing understanding of own situation	Poor coping style
Teaching the sick-listed employee to cope with his disabilities	Character style
Positive personal characteristics of the employee	Negative Illness perceptions
Taking employee seriously	Negative attitude towards work resumption
A good occupational physician	Social influence
Providing RTW vocational rehabilitation as soon as possible	Negative self-efficacy expectations
Positive social environment	Inefficient guidance from RTW stakeholders
Support from colleagues	Inefficient coping style
Influencing thoughts/behaviour	Task contents
Positive meaning of work	Problematic working environment
Financial incentives for employee	Problematic work relationships
Financial incentives for employer	Adverse workplace conditions
Communication at the same level or in the same language	Combined workload
Positive illness perceptions	Impairment
Positive workplace conditions	Imbalanced work ability task contents
Open communication between RTW stakeholders	History of sickness absence
Optimal guidance from vocational rehabilitation professionals	Lack of social support
Cooperation between all RTW stakeholders	
Cooperative vocational rehabilitation by professional-social network of employee	
Improving social skills of employee	
Encouraging sense of responsibility	
Confronting employee with his own future	

## Appendix 2

See Table 3.

**Table 3:** Factors that hinder RTW of long-term sick-listed employees according to 80 % of the respondents (*n* = 103)

Factors that hinder RTW	Percentage (%)
Inefficient coping style	91
Negative illness perceptions	89
Secondary gain from illness	89
Treating physicians that promote illness behaviour or advise incorrectly concerning RTW	88
Inefficient guidance from different RTW stakeholders	86
Medicalising	82
Negative attitude from employee towards work resumption	81
Physicians focussing on strictly medical issues instead of paying attention to non-medical factors	80

## Appendix 3

See Table 4.

**Table 4:** Factors that promote RTW of long-term sick-listed employees according to 80 % of the respondents (*n* = 103)

Factors that promote RTW	(%)
Influencing thoughts/behaviour	96
Positive attitude towards work resumption	94
Positive illness perceptions	90
Motivation of sick-listed employee to RTW	92
Effective communication with sick-listed employee	91
Increasing understanding of own situation	92
Teaching the sick-listed employee to cope with his/her disabilities	91
Positive personal characteristics of the employee	90
Avoiding conflicting advice of treating physicians	90
Taking employee seriously	90
A good occupational physician	89
Positive self-efficacy	88
Interest of treating physicians for work issues	85
Providing RTW vocational rehabilitation as soon as possible	83
